# Validation of cost-efficient EEG experimental setup for neural tracking in an auditory attention task

**DOI:** 10.1038/s41598-023-49990-6

**Published:** 2023-12-19

**Authors:** Jiyeon Ha, Seung-Cheol Baek, Yoonseob Lim, Jae Ho Chung

**Affiliations:** 1 Department of HY-KIST Bio-Convergence, Hanyang University, Seoul, 04763 Korea; 2 Center for Intelligent & Interactive Robotics, Artificial Intelligence and Robot Institute, Korea Institute of Science and Technology, Seoul, 02792 Korea; 3 Department of Otolaryngology-Head and Neck Surgery, College of Medicine, Hanyang University, Seoul, 04763 Korea; 4 Research Group Neurocognition of Music and Language, Max Planck Institute for Empirical Aesthetics, 60322 Frankfurt\ Main, Germany; 5 Department of Otolaryngology-Head and Neck Surgery, School of Medicine, Hanyang University, 222-Wangshimni-ro, Seongdong-gu, Seoul, 133-792 Korea

**Keywords:** Mathematics and computing, Neuroscience, Auditory system, Sensory processing

## Abstract

When individuals listen to speech, their neural activity phase-locks to the slow temporal rhythm, which is commonly referred to as “neural tracking”. The neural tracking mechanism allows for the detection of an attended sound source in a multi-talker situation by decoding neural signals obtained by electroencephalography (EEG), known as auditory attention decoding (AAD). Neural tracking with AAD can be utilized as an objective measurement tool for diverse clinical contexts, and it has potential to be applied to neuro-steered hearing devices. To effectively utilize this technology, it is essential to enhance the accessibility of EEG experimental setup and analysis. The aim of the study was to develop a cost-efficient neural tracking system and validate the feasibility of neural tracking measurement by conducting an AAD task using an offline and real-time decoder model outside the soundproof environment. We devised a neural tracking system capable of conducting AAD experiments using an OpenBCI and Arduino board. Nine participants were recruited to assess the performance of the AAD using the developed system, which involved presenting competing speech signals in an experiment setting without soundproofing. As a result, the offline decoder model demonstrated an average performance of 90%, and real-time decoder model exhibited a performance of 78%. The present study demonstrates the feasibility of implementing neural tracking and AAD using cost-effective devices in a practical environment.

## Introduction

In recent years, there has been a growing interest in the brain’s ability to track the slow temporal rhythms found in acoustic features such as the stimulus envelope or linguistic information like syllables, words and speech^1–4^. The tracking of neural activity to these temporal rhythms is often referred to as “neural tracking”. Such a neural tracking framework enables the versatility of providing insights into a wide range of auditory and linguistic processes^5,6^.

In particular, by decoding neural responses associated with the speech envelope, many researchers aim to unravel the underlying mechanisms involved in speech processing in the central auditory pathway based on electroencephalography (EEG). For example, several studies have demonstrated a strong correlation between neural tracking with the speech comprehension^7,8^, as well as various disorders, including attention-deficit/hyperactivity disorder^9^ and phonological deficits^10^. These studies showed the potential of neural speech tracking as an objective diagnostic tool for diverse clinical contexts^6^.

Furthermore, an auditory attention decoding (AAD) technique has been introduced, which enables the detection of the speech signal that the listener is focusing on in a multi-talker scenario^11–13^. This technique utilizes neural tracking to reconstruct the envelope of the attended speech information and allows for the selection of the speech signal with the highest correlation to the envelope of the attended speech signal, by enabling the assessment of auditory attention^14–16^. Based on these AAD features, the study of EEG-based AAD has implications beyond basic research, extending to practical applications in the audiology field including neuro-steered hearing devices^17–19^. Neuro-steered hearing devices would enable listeners to selectively amplify desired sounds based on the decoded neural responses, thereby greatly enhancing speech comprehension and communication in challenging listening environments. In addition, to broaden the application of neural tracking to diverse clinical contexts, it is important to obtain a large neural tracking dataset that encompasses individuals of diverse ages and includes a variety of auditory pathologies.

However, most EEG-based neural tracking and AAD studies have primarily been conducted in laboratory-based settings with soundproof and electrically shielded rooms^7,11, 18, 20^. In particular, the use of complex and expensive EEG experimental setups limits the accessibility of neural tracking technologies. In addition, the strict control of experimental conditions can pose challenges for certain populations, including individuals with locked-in syndrome and older adults, due to the extended duration of the experiments and limited space available. In this regard, simplifying the EEG experimental setup is an essential step in improving the accessibility of neural tracking measures in many areas of clinical application. Recently, there have been several studies to enhance portability of EEG systems, including the use of miniature EEG electrodes, such as cEEGrid^21^, in-ear EEG^19^ and mobile EEG systems^22^. However, for most neural tracking experiments, it is necessary to employ an additional system capable of accurately synchronizing sound onset triggers with EEG signals as well as the EEG system. Therefore, efforts should be made to minimize the overall experimental setup burden associated with both EEG and the synchronizing system. So far, various studies have attempted to perform auditory attention tasks, such as the oddball task using two different tone sounds, with a portable or cost-efficient EEG experimental setup^23,24^. They have shown meaningful classification performance for the auditory attention to one tone sound based on event-related potential, suggesting that the auditory attention of listeners can be effectively detected using a cost-efficient EEG setup. However, given that they used simple tone sounds as stimuli, which are quite different from sounds commonly heard in daily life, there is still a need to explicitly validate the feasibility of neural tracking with continuous speech stimuli under the cost-effective EEG experimental setup. Furthermore, the ability to perform real-time tracking with minimal computational load would greatly enhance the practicality and accessibility of neural tracking or AAD technologies.

To this end, the aim of the study was to develop a cost-efficient neural tracking system capable of tracking neural activity in response to auditory signals using EEG at a lower cost. The performance and usability of the developed system were validated using a real-time AAD experiment based on a well-established two competing speaker paradigm in a practical environment^11,18^.

## Methods

### Design of a cost-efficient neural tracking system

**Figure 1:**
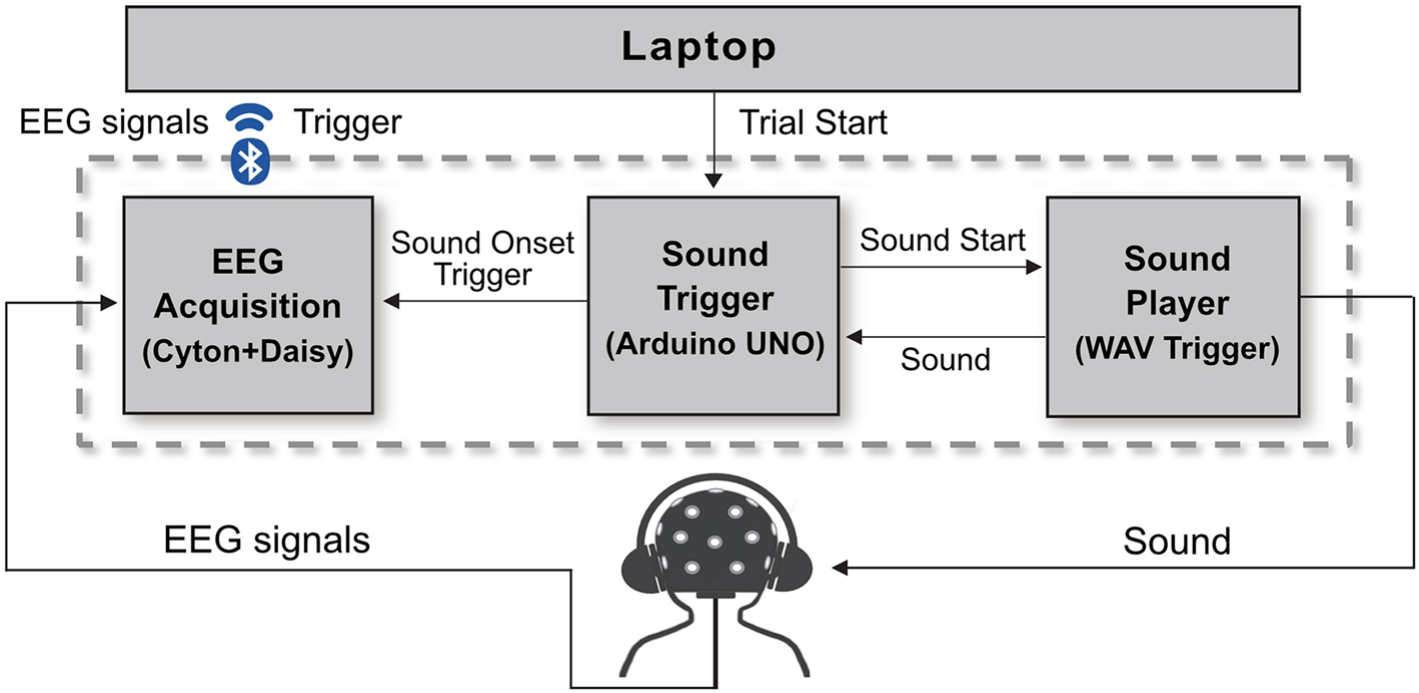
Block diagram of the cost-efficient neural tracking system consisting of the EEG acquisition module, the sound trigger module, and the sound player module.

A block diagram of a Cost-efficient Neural Tracking System is shown in Fig. 1. To perform an auditory task for neural tracking, EEG signals were recorded with precise timing information about the onset of audio, enabling the capture of corresponding EEG signals for speech stimuli. Consequently, the proposed system comprises an EEG acquisition module, a sound player module, and a sound trigger module (Fig. 1). The details of the three modules are described below.

**Figure 2:**
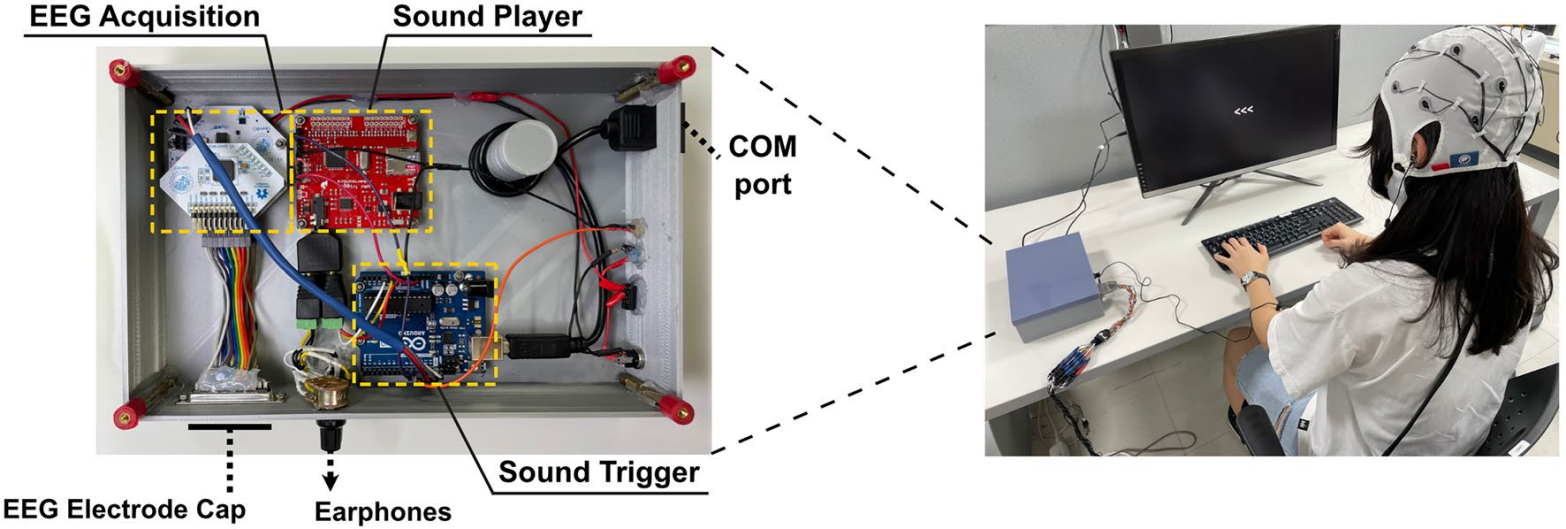
The Cost-Efficient Neural Tracking System: The photograph on the left showcases the inside of the system, which is composed of three modules. On the right, there is a photograph depicting a participant using the cost-efficient neural tracking system to perform an auditory attention decoding task.

#### The EEG acquisition module

The current study utilized an OpenBCI board (Cyton with Daisy Biosensing Boards, OpenBCI, USA) for the EEG acquisition. OpenBCI boards have been validated in various studies and are known for their low-cost and high-quality performance^25–27^. These boards can acquire both EEG signals and external trigger signals that contain precise time information for synchronizing sound onset times with the corresponding EEG signals. To ensure accurate synchronization, the EEG acquisition module captures the EEG signals and the sound onset trigger simultaneously, minimizing any potential timing discrepancies. The acquired data can then be wirelessly transmitted to a laptop through a Bluetooth connection. Open-source data streaming software like Lab Streaming Layer (Kothe C. Lab Streaming Layer) and Brainflow (Parfenov, A BrainFlow) can be utilized for data streaming and analysis.

#### The sound trigger module

For the sound trigger module, an Arduino UNO board was employed. The Arduino UNO board is easily programmable and capable of interacting with various electronic devices, including computers. When initiating the presentation of stimuli, the laptop sends a command to the sound trigger module during the auditory task. Upon receiving the command, the sound trigger module instructs the sound player module to play the speech sound. Furthermore, by monitoring the analog voltage level of the audio output from the sound player module, the sound trigger module detects the precise time when the sound begins. It generates a trigger signal, referred to as the sound onset trigger in Fig. 1, which serves as a marker to identify the onset of the speech stimulus.

#### Sound player module

A WAV trigger (WAV Trigger, Robertsonics, CA, USA) was used as the sound player module. When the sound player module received a command signal from the sound trigger module, it played .wav files saved on a micro-SD card. Additionally, to establish a timestamp of sound onset, the sound player module simultaneously delivered a sound signal to both the sound trigger module and the subject.

Figure 2 shows the inside of the cost-efficient neural tracking system consisting of three modules, and a subject performing the auditory task with the devised system. During the auditory task, the system is placed next to the participant and connected to the earphones, EEG electrode cap, and COM port. The EEG electrode cap is connected to the EEG acquisition module through a parallel port, and the COM port allows the sound trigger module to interact with the laptop. A potentiometer (placed above the earphone adapter) can be used by the participant to regulate the sound level.

### The decoder model

In order to evaluate the practicality of the developed cost-efficient system for neural tracking, we performed the AAD experiment, which enables objective measurements of neural tracking. The current study employed two distinct decoder models: an offline decoder^11^ and a real-time decoder^28^. The offline decoder was utilized after collecting all data from the AAD task, whereas the real-time decoder was employed to observe decoder results in real time during the AAD task.

#### Offline decoder model

The offline decoder $D(\tau ,n)$ was used to reconstruct the envelope of attended speech stimulus using the post-stimulus EEG signals based on a ridge linear regression^29^. We represent the EEG signals of the electrode *n* at time t = 1 … T as $R(t,n)$. The reconstructed speech envelope $\widehat{S}$ is given by:

1 $$\widehat{S}\left(t\right)= \sum_{n}\sum_{\tau }D\left(\tau ,n\right)R\left(t-\tau ,n\right).$$

where $\widehat{S}(t)$ represents the reconstructed speech envelope at a given time t. A timelags τ models the delay of EEG signals in response to speech. The decoder model *D* is optimized by the least square method such that it minimizes the difference between the envelope of attended speech *S* and the reconstructed envelope $\widehat{{\text{S}}}$ estimated by the corresponding EEG signal across all channels and time-lags.

2 $$D ={(R{R}^{T}+\lambda I)}^{-1}R{S}^{T}$$

where λ and $I$ denote a regularization parameter and the identity matrix, respectively. Based on a previous study^28^, the regularization parameter λ and the time lags were set at 10 and 0–250 ms post-stimulus, respectively.

The performance of the offline decoder model was evaluated by Pearson's correlations between the reconstructed speech envelopes (Eq. (1)) and the actual attended and unattended speech envelopes. If the envelope correlation (r) with the attended speech envelope for each trial was greater than that with the unattended speech envelope, the reconstructed speech envelope had been correctly evaluated. Decoder accuracy was estimated as the percentage of correctly evaluated trials.

#### Real-time decoder model

To carry out the real-time AAD, we used a real time linear decoder model suggested by a prior study^28^. To operate the decoder in real time, a sliding window that extracts a snippet of the EEG signal and an envelope of the speech stimulus in a given interval was applied to the offline decoder model. The reconstructed speech envelope $\widehat{{S}_{i}}$ is given by:

3 $$\widehat{{S}_{i}}\left({t}_{i}\right)= \sum_{n}\sum_{\tau }\overline{D }\left(\tau ,n\right){R}_{i}\left({t}_{i}-\tau , n\right).$$

A real-time decoder $\overline{D }$ can also be formalized as a function that maps snippets of EEG signals from *n* channels, denoted as ${R}_{i}({t}_{i}-\tau , n)$, to the corresponding snippets of speech envelopes, ${S}_{i}$, at time ${t}_{i}$. Given a speech signal of length T, ${t}_{i}$, a subset of *t* = 1,⋅⋅⋅, T, spans from (i−1)M − W + 1 to (i−1)M. Here, W denotes the width of the window, and M is the moving interval of the window. Here, as mentioned, snippets are extracted by the sliding window, and the subscript *i* denotes the snippet from the *i*-th window.

The real-time decoder $\overline{D }$ was built by averaging all snippet-wise decoders ${D}_{i}$ over all trials for the decoder model construction (i.e., the training set). The snippet-wise decoder ${D}_{i}$ can be estimated by solving the formula based on least squares method as follows:

4 $${D}_{i}={({R}_{i}{R}_{i}^{T}+ \mathrm{\lambda I})}^{-1}{R}_{i}{S}_{i}^{T}.$$

Consistent with the offline decoder model, the λ and τ were selected 10 and 0-250 ms post-stimulus, respectively. In addition, W and M were chosen as 15 s and 1 s, respectively, as proposed by the previous study^28^.

To estimate performance of the real-time decoder, we compared the reconstructed snippet-wise speech envelopes using the real-time decoder for each subject (Eq. (3)) to actual speech envelopes over trials to evaluate the decoder model (i.e., the test set). This comparison followed the same approach as the offline decoder model, but there is a difference caused by the application of sliding window. Accordingly, each trial produces multiple envelope correlation, leading to multiple assessments that compare the reconstructed speech envelope with actual speech envelopes for each trial. That is, the real-time decoder accuracy was estimated as the percentage of correctly evaluated windows over all test set.

### The exponential moving average

If the envelope correlations (i.e., Pearson's correlations) in a trial fluctuate excessively, this can lead to unstable decoding results. Therefore, to smooth out envelope correlation fluctuations, we applied an exponential moving average (EMA)—a type of moving average that places greater weight on the most recent data points—to the envelope correlations related speaker 1 and speaker 2, respectively. The EMA-applied envelope correlation in the *i*-th window can be estimated as:

5 $${\widehat{r}}_{i}= \left\{\begin{array}{c}{ r}_{i} , \,\,\,\,\,\,\,\,\,\,\,\,\,\,\,\,\,\,\,\,\,\,\,\,\,\,\,\,\,\,\,\,\,\,\,\,\,\,\,\,\,\,\,\,\,\,\,\,\,\,\,\,\,\,\,\,\,\,\,\,i=1\\ \alpha \cdot {r}_{i}+\left(1-\alpha \right)\cdot {\widehat{r}}_{i-1} , \,\,\,\,\,\,\,\,\,i>1\end{array}\right.$$

where ${r}_{i}$ is the envelope correlation of the *i*-th window. The weight α determines the extent to which the calculation of the EMA statistics is influenced by older data. Based on previous studies^30,31^ suggesting weight values ranging from around 0.1 to 0.3, the weight α was determined by 0.1.

### Auditory attention decoding experiment

#### Participants

For the current experiment, a total of 10 participants were recruited, ranging in age from 25 to 34, with 2 females. One participant was excluded due to poor results in the behavior test, resulting in the analysis being conducted on data from nine subjects. All participants were right-handed native Korean speakers. Individuals with a history of neurological conditions or hearing loss were excluded from the study. Prior to participating, all participants provided informed consent. The experiment adhered to the ethical standards outlined in the Declaration of Helsinki and received approval from the institutional review board of the Korea Institute of Science and Technology (IRB number: 202207–006).

#### Stimulus

The speech stimulus utilized in the experiment consisted of excerpts from two stories, namely "Twenty Thousand Leagues Under the Sea" and "Journey to the Center of the Earth" by Jules Verne. Each speech excerpt was recorded in Korean by a different male speaker and divided into 30 trials, with each trial lasting for 1 min. To ensure continuous auditory stimulation and prevent a loss of concentration, speech pauses longer than 0.5 s were reduced to 0.5 s. For the AAD task, the two speech stimuli were delivered dichotically using low-cost conventional in-ear earphones (P235, Britz, Korea). The individual sound intensities were adjusted to comfortable levels for each participant.

#### Procedure

The AAD experiment was carried out in a meeting room with a noise level of 46–47 dB. The meeting room was not acoustically isolated, resulting in audible background noise such as footsteps, door sounds, and conversation. Furthermore, there was no electrical shielding implemented. Participants were seated in front of the monitor in a comfortable chair. The current experiment was a modified version of the experimental procedure used in the real time AAD experiment, which was based on a dichotic listening paradigm^28^. Participants listened to two competing speeches presented dichotically, with one speech played on the left side and the other on the right side. They were asked to focus their attention solely on one of the speeches (speaker 1) while disregarding the other speech (speaker 2).

**Figure 3:**
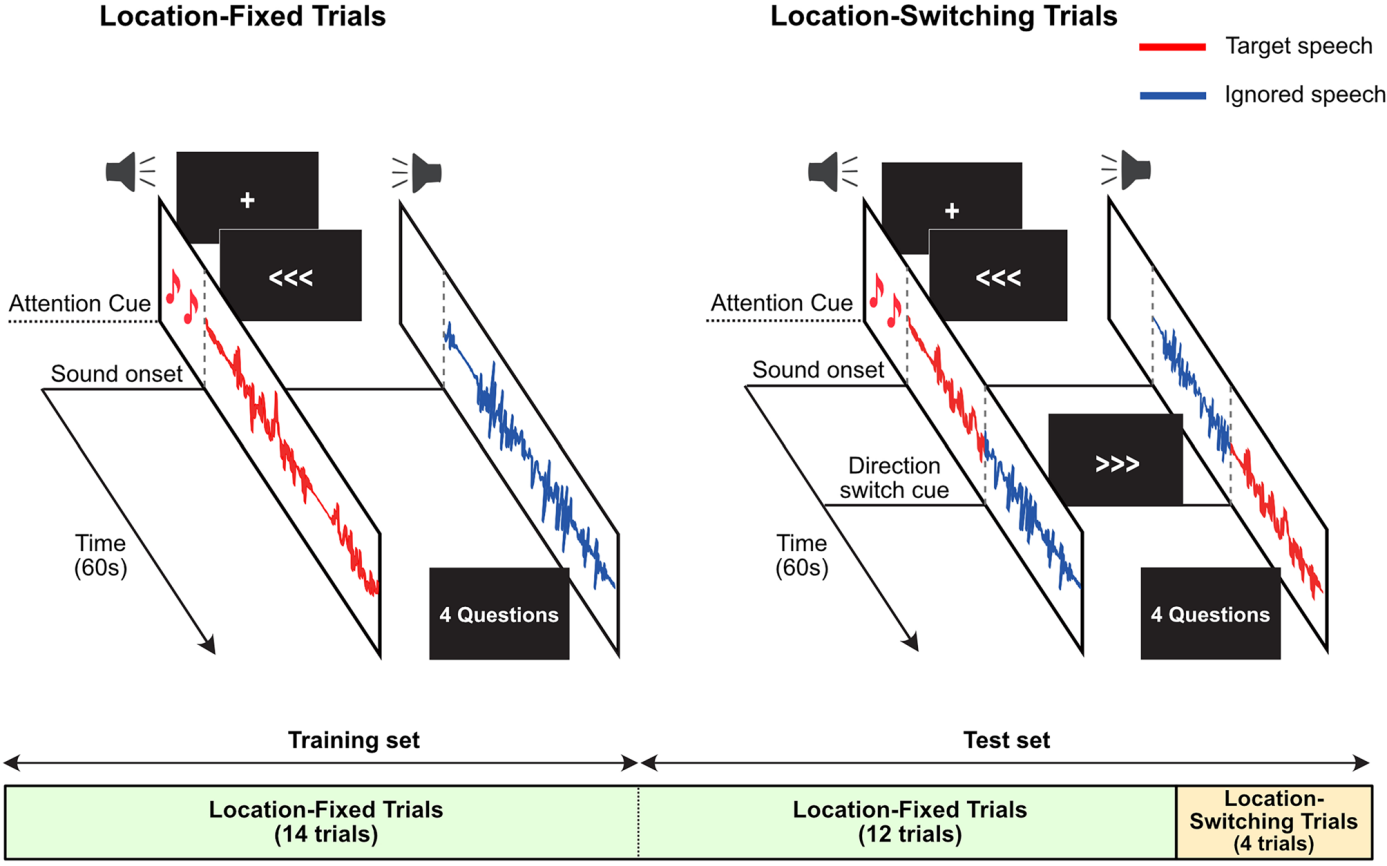
Illustration of the experimental procedure. The experimental design consists of location-fixed trials (left) and location-switching trials (right). The attention cue indicates the onset of the directional sound, helping identify the direction of attention in each trial. For location-switching trials, the direction of the target speech changed at the direction switch cue near the middle of the speech stimuli. In each location-switching trial, the direction switch cue was presented at a slightly different time point. The under panel shows an overview of an AAD task session, which includes the training set of 14 trials and the test set of 16 trials.

The AAD experiment consists of 30 trials, and each trial lasts for 1 min. To avoid a directional bias, the side to which the target speech was delivered was pseudo-randomly determined (i.e., 15 trials to the left and 15 to the right). Out of the 30 trials, 26 trials were location-fixed trials, while the remaining 4 trials were location-switching trials, as shown in Fig. 3. In location-fixed trials, the direction of attention towards the sound was fixed throughout the trial, while in location-switching trials, the direction of the target speaker changed to opposite ear during the trial. Participants maintained their focus on the same speaker during location-switching trials.

Two types of attention cues were provided to indicate the direction participants should focus on: directional sounds, which were tone sounds presented twice at the beginning of each trial and lasted for 500 ms, and a directional arrow displayed on a monitor (Fig. 3). The directional sound was presented exclusively on the side corresponding to the intended direction of attention. Simultaneously, the directional arrow pointed in the same direction and remained displayed on the monitor throughout the duration of the stimuli. Participants were able to perceive when the direction of attention for the sound indicated by the directional arrow switched, and they had to subsequently adjust the direction of their focused attention accordingly. The direction of the target speaker changed at a direction switch cue near the middle of the speech stimuli, and we introduced small jitters at the time the attention switch cue appeared in each trial.

To train and validate the offline decoder model, only 26 location-fixed trials were used. We considered the location-switching trials as inappropriate for inclusion in the evaluation of offline AAD performance because of the occurrence of speaker location change within a single trial. The speech envelope of each trial was reconstructed using averaged weight of the offline decoders trained on all other trials (25 trials) of each subject, known as a leave-one-out cross-validation approach.

For the real-time decoder model, the first 14 location-fixed trials were used to construct the real time decoder model (the training set), while the rest of the trials (12 fixed trials and 4 switching trials) were used to evaluate the performance of the decoder (the test set) as mentioned in the section *Real-time Decoder Model.*

The decoder model was individually trained in real time for each participant during the presentation of the training set, and its performance was evaluated while executing the test set. No additional time was required for creating the decoder between the training set and the test set. At the end of each trial, subjects were asked to complete four different questions to ensure their attention: two were related to the speaker 1 and two for the speaker 2.

#### Data acquisition & analysis

The EEG signals were recorded using a 19-electrode Ag/AgCl EEG cap provided by OpenBCI. For the better portability of the devised system, we used a small number of EEG electrodes with the OpenBCI board. To this end, we referred to the previous study showing that good AAD performance could be achieved by 15 EEG electrodes^18^. Accordingly, we selected only fifteen EEG channel locations (Fz, Cz, C3, C4, P7, P8, Pz, F7, F8, F3, F4, T7, T8, P3, and P4, according to the International 10/20 System) on the basis of the findings that the best electrode sites for the AAD included the regions around the temporal and front-central lobes^32,33^. An electrode at CPz served as reference during the data acquisition.

During the experiment, EEG signals were sampled at 125 Hz and were continuously transmitted to the laptop by BrainFlow without preprocessing. After the onset of speech stimuli, acquired EEG signals were accumulated in a buffer for the first 15 s. Analysis was then conducted starting from the point when 15 s of data had been accumulated. During the AAD task, the EEG signals in the buffer were updated every second with the most recent EEG signals, and all data analyses from EEG preprocessing to decoder training or testing were performed with a time resolution of 1 s.

Afterwards, the accumulated EEG signals were re-referenced to a common average reference and filtered using a passband of 0.5–8 Hz. The filtering range was determined by an AAD simulation based on the dataset from a previous study^28^ in which AAD performance over various filtering ranges was compared (details can be seen in Supplementary Information). Although previous studies of EEG-based AAD have generally not included the 0.5–2 Hz band in their analyses^18,28, 34^, we noticed that including the 0.5–2 Hz band improved decoder accuracy (see Supplementary Information). Subsequently, the EEG signals were down-sampled to 64 Hz and z-scored. All preprocessing was performed in real time for every snippet of EEG signal (as mentioned in section *Real-time Decoder Model*). Raw speech envelopes were extracted by taking the absolute values of analytic speech signals (by Hilbert transform) and bandpass-filtered in the same way as the EEG signals, followed by down-sampling (to 64 Hz) and z-scoring.

To streamline the experiment, we preprocessed the speech signals by extracting their envelopes and storing them in the laptop. We also extracted corresponding segments from the stored speech envelopes during the EEG signal preprocessing for training and attention decoding.

All data analyses were implemented using a custom-made Python script (that is available at our GitHub address), and the laptop with a central processing unit (CPU, 2.6 GHz, i7-9740H, Intel(R) Core (TM)) and random access memory (RAM, 32 GB).

### Temporal sensitivity of neural tracking to changes in spatial attention

To assess how effectively the devised system responds to sudden changes in the spatial location of the target speaker, we measured the temporal sensitivity of the neural tracking for the spatial change. This temporal sensitivity was derived by time required to achieve precise decoding outcome for changes in the direction of the target speech in four location-switching trials. For the analysis of temporal sensitivity, we used the raw envelope correlation values without applying exponential moving average. To rule out cases where the correct outcome occurred momentarily by chance, we considered only genuine spatial changes when the correct decoding result persisted for more than 5 s. Also, in cases where the correct decoding outcome was not obtained until the end of the trial, the response time was calculated as from the direction switch point to the end of the trial.

## Results

### Behavior results

The behavioral results showed that participants attended well to the target speaker during the AAD task while ignoring the other. The nine participants correctly answered 91.11 ± 1.11% of the questions on the attended speech. Conversely, the correct answer rate for the ignored speech was 26.11 ± 1.69%, which is lower than the upper bound of the chance level (35%) determined by the binomial test at a significance level of 5% (n = 60, p = 0.25).

### Offline AAD performance

**Figure 4:**
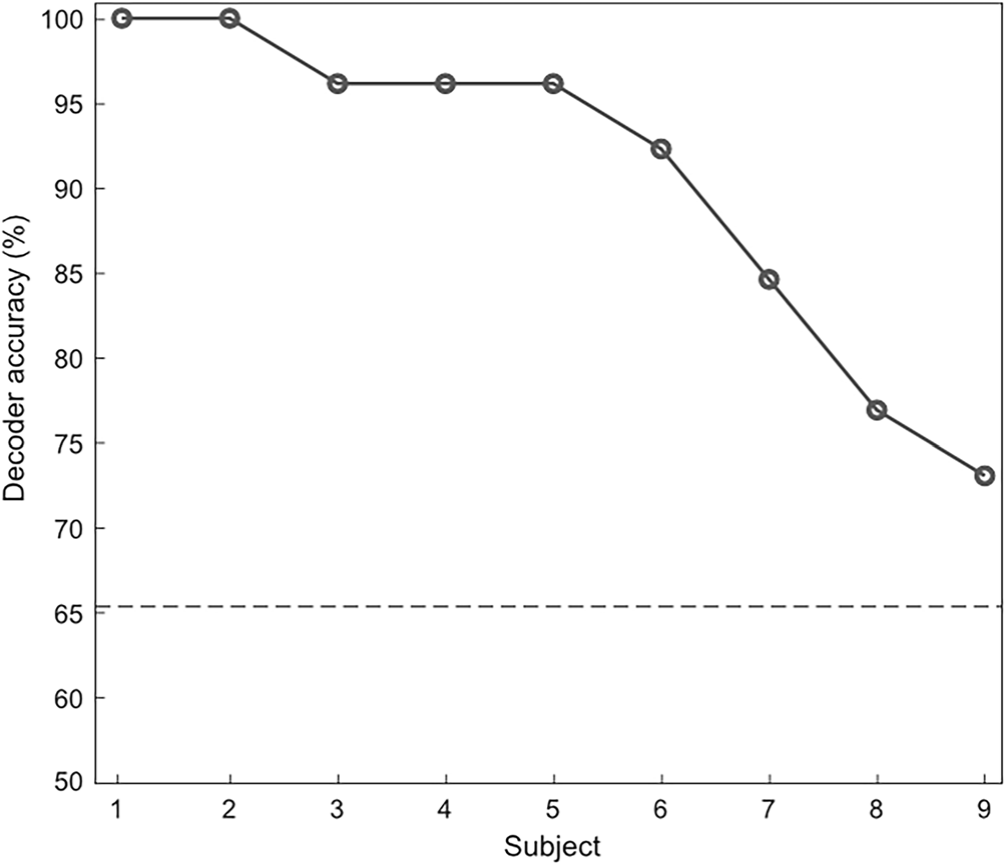
Results of the AAD performance for the offline decoder. For the offline decoder, the decoder accuracy was evaluated using only location-fixed trials (26 trials) based on leave-one-out cross-validation. The dashed line indicates the chance level (65.38%) based on a binomial test at the 5% significance level.

The performance of the offline decoder was evaluated using only location-fixed trials through leave-one-out cross-validation (26 trials). Figure 4 shows individual offline decoder accuracy. The individual decoder accuracies for all subjects were significantly above chance level (65.38%, n = 26, p = 0.5). And the average offline decoder accuracy was 90.60 ± 3.34% across 9 subjects.

### Real-time AAD performance

**Figure 5:**
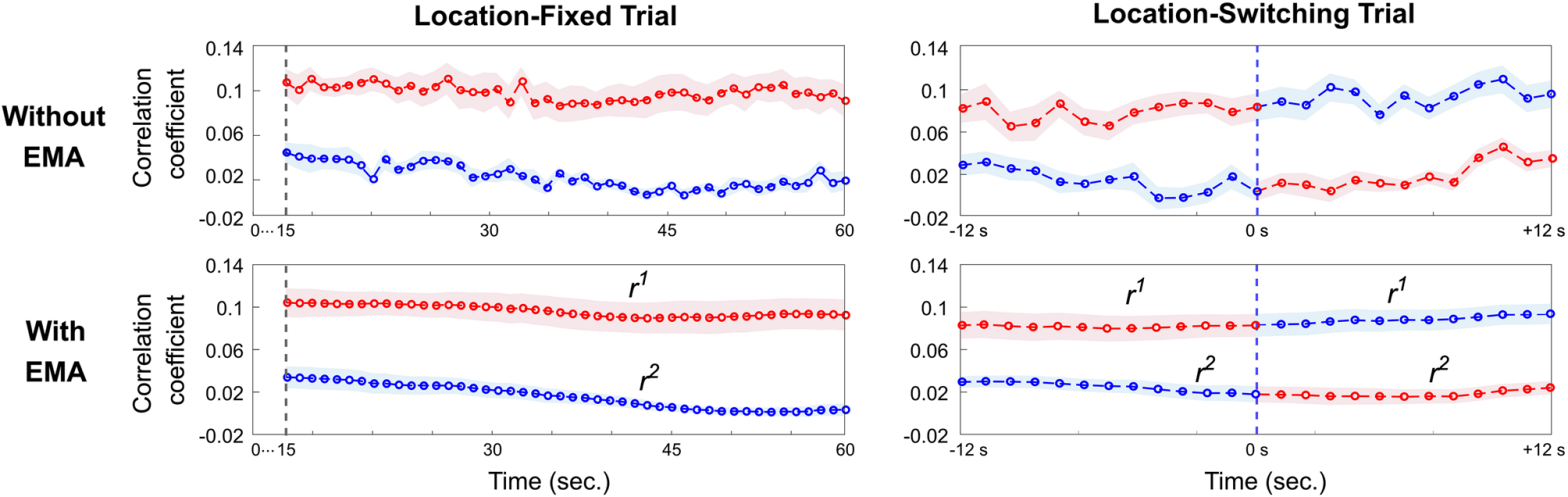
A grand average of envelope correlation traces for the real-time decoder. To smooth fluctuations in envelope correlations, exponential moving average (EMA) was employed, giving higher weight to the most recent data points for both the envelope correlations associated with the speaker 1 (the target speaker, indicated by r^1^) and the speaker 2 (indicated by r^2^). The top of the graph are traces without applying EMA, while the bottom are the traces with EMA applied. The graphs on the left are traces from a location-fixed trial, and on the right, from a location-switching trial. In the location-switching trial, each trial was aligned based on its respective switching point (denoted as 0 s on the time axis), and a time window of 12 s before and after the direction switch point (blue dashed line) was selected. The colors of line are used to distinguish the direction in which speech is presented, and the direction of speech was distributed with an equal number of trials on both the left and right sides. The shaded areas indicate the standard error of the mean across subjects.

**Figure 6:**
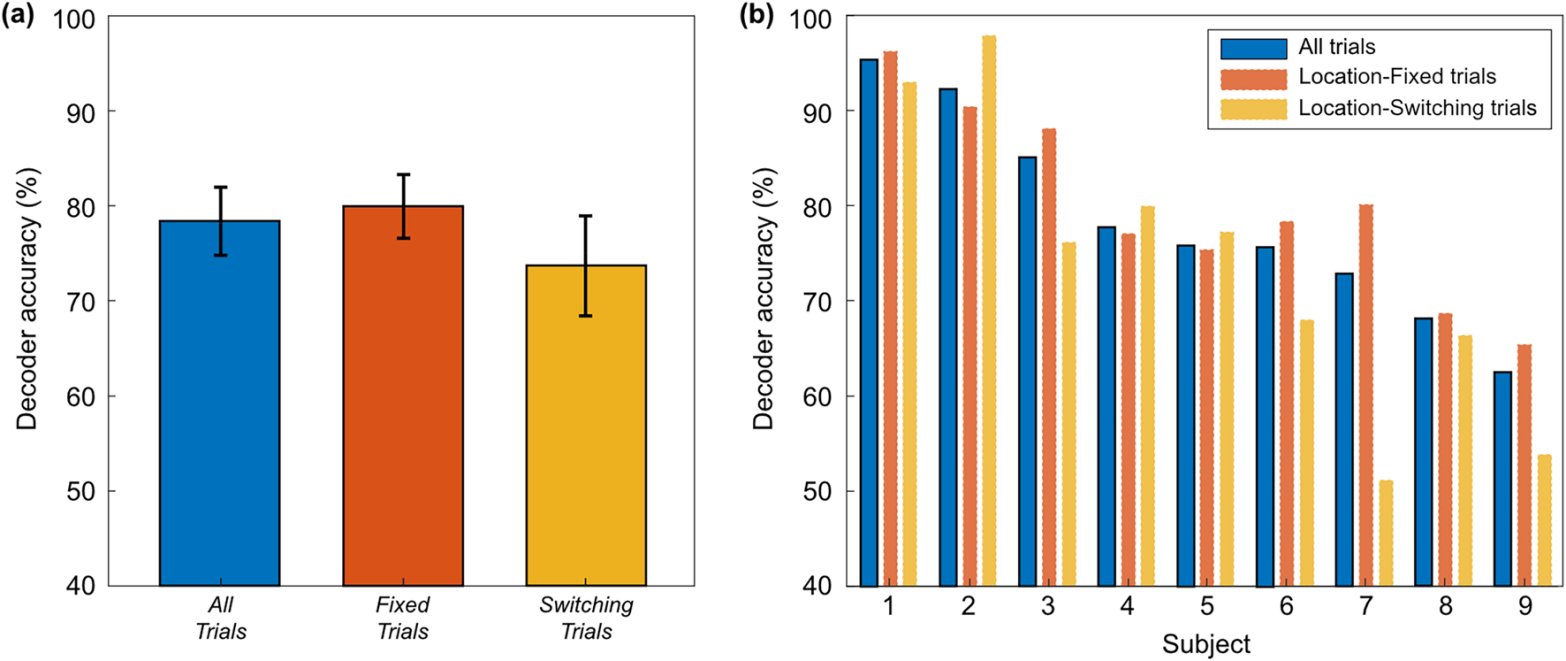
Results of AAD performance for the real-time decoder. (**a**) Average decoder accuracies across all participants: all 16 trials (blue), the 12 location-fixed trials (red), and the 4 location-switching trials (yellow). The black line on each bar denotes the standard error of the mean. (**b**) Individual decoder accuracies for the nine volunteers.

Figure 5 shows a grand average of envelope correlation traces (*r*) with standard error of the mean (SEM). After applying the EMA, the traces became smoother, and the AAD performance improved. As shown in Fig. 5, the decoder model could track participants’ auditory attention quite well in both fixed and switching trials. In order to visualize location-switching trials with different switching points (between 27 and 33 s) collectively, each trial was aligned based on its respective switching point, and a time window of 12 s before and after the switching point was selected. The average value and SEM were then calculated within this time window. The average decoder accuracy for all 16 test trials was 78.37 ± 2.03%. For the 12 location-fixed trials, it was 79.92 ± 3.36%. For the 4 location-switching trials, it was 73.67 ± 5.27%, slightly lower than for the location-fixed trials (Fig. 6). These results show that our cost-efficient experimental setup for real-time neural tracking and AAD works well in both location-fixed and location-switching trials.

### Temporal sensitivity of neural tracking to changes in spatial attention

The temporal sensitivity to changes in spatial attention was measured as an average of 7.97 ± 1.56 s across nine subjects in four location-switching trials (total 36 trials). In detail, in 18 out of 36 trials, the temporal sensitivity was measured as 1 s, indicating that the devised system can successfully perform AAD and neural tracking even when the listener’s spatial attention changes suddenly. However, in three trials, the correct outcome was not obtained until the end of the trial. The temporal sensitivity in these cases was recorded as 30 s. The individual temporal sensitivity results can be seen in the supplementary material (Supplementary Table S1).

## Discussion

The present study aimed to validate the feasibility of the cost-efficient system for neural tracking in non-soundproof environment, with the goal of enhancing the applicability and accessibility of neural tracking in clinical contexts. To this end, we performed the AAD task which is based on neural tracking mechanism and achieved the offline decoder performance of about 90% and the real-time decoder performance of about 78%. Moreover, the devised system could effectively decode the listener's auditory attention, even in the event of a sudden shift in spatial attention.

In the current study, the AAD task was conducted using the laptop and devised cost-efficient system with 15 EEG electrodes in the normal meeting room. Accordingly, this study employed a different experimental setup involving a low-cost EEG system with a reduced number of EEG electrodes compared to a previous experiment^28^. While the same decoder model and AAD paradigm were employed, the present study took place in a practical environment without soundproofing. Despite the cost-saving measures, the present work achieved an acceptable real-time decoder performance (about 78%), which can be compared to the earlier study that reported a performance of around 70% using the same decoder model^28^. In addition, real-time decoder's performance with the devised system is comparable to other real-time AAD studies, as it reports a decoder accuracy of approximately 80%^35,36^. Furthermore, the offline decoder accuracy achieved in this study, approximately 90%, is also comparable to other lab-based studies, such as O'Sullivan et al.^11^, which reported a performance of about 89% with 40 subjects^11^.

To explore the possibility of neural tracking measurement in diverse listening conditions, many studies have addressed the spatial change in auditory attention and demonstrated good performance^37–40^. In the current study, we assessed the temporal sensitivity to spatial change in the target speaker within four location-switching trials where the listener remained focus on the same speaker even when the direction of attention changed. This scenario partially reflects daily life situations where the target speaker being in moving or when the listener swiftly turning their head. To make an objective comparison with high-end EEG setups, we followed the same experimental paradigm used in a previous study^28^. As a result, the average temporal sensitivity was found to be approximately 8 s, which seems to demonstrate the feasibility of the devised system in different listening conditions. Nevertheless, it is important to note that this study was limited to only four location-switching trials in examining changes in the spatial location of attention, emphasizing the need for further validation through additional experiments. Furthermore, future research should aim to extend the applicability of neural tracking to real-world scenarios, including dynamic listening conditions where the attended speaker switches.

In recent studies, several techniques that employ AAD for neural tracking have been introduced. These methods utilize non-linear or non-supervised training approaches and have demonstrated good performance^41–45^. Nevertheless, compared to linear decoder models used in this study, these approaches may impose higher computational demands, potentially necessitating high-performance computing resources. Moreover, the decoder model relies on the acoustic envelope of original sound sources to compute the envelope correlation with the estimated envelope derived from neural recordings. However, in real-world listening scenarios, multiple sounds and background noises coexist with the original speech, necessitating a speech separation process to distinguish individual speech sounds from audio mixtures before employing AAD^34,46^. This additional step often entails a higher computational workload. From this perspective, it is important to carefully balance performance and usability to propose a more suitable approach for real-time neural tracking that aligns with the objectives of AAD.

Neural tracking measurements are considered as a versatile tool that can be measured in various parts of the auditory system^3–5^. The current results, which demonstrate reasonable performance in measuring neural tracking in a cost-effective setup, could help to efficiently assess auditory and linguistic processing as a diagnostic tool. Moreover, the utilization of the devised cost-efficient system to perform real-time neural tracking might open possibilities for expanding to a wider range of clinical applications.

## Conclusions

To validate the feasibility of implementing neural tracking in a cost-efficient experimental setup, the present study developed a cost-efficient system capable of neural tracking and evaluate its performance by conducting AAD tasks in a non-soundproof environment. The present study demonstrated promising performance in both the offline and real-time decoders, achieving approximately 90% and 78% decoder accuracy, respectively. Moreover, the decoder's ability to track the attended speech envelope demonstrated results comparable to studies conducted in laboratory conditions, thus suggesting the promising applicability of neural tracking in a practical environment.

### Supplementary Information

Supplementary Information 1.

## Data Availability

All the methods used to implement the cost-efficient system for neural tracking are available at: https://github.com/HYAuditory/System-for-Neural-Tracking.

## References

[1] Shannon RV, Zeng F-G, Kamath V, Wygonski J, Ekelid M (1995). Speech recognition with primarily temporal cues. Science.

[2] Ding N, Simon JZ (2012). Neural coding of continuous speech in auditory cortex during monaural and dichotic listening. J. Neurophysiol..

[3] Aiken SJ, Picton TW (2008). Human cortical responses to the speech envelope. Ear Hear..

[4] Daube C, Ince RAA, Gross J (2019). Simple acoustic features can explain phoneme-based predictions of cortical responses to speech. Curr. Biol..

[5] Gillis M, Kries J, Vandermosten M, Francart T (2023). Neural tracking of linguistic and acoustic speech representations decreases with advancing age. NeuroImage.

[6] Gillis M, Canneyt JV, Francart T, Vanthornhout J (2022). Neural tracking as a diagnostic tool to assess the auditory pathway. bioRxiv.

[7] Vanthornhout J, Decruy L, Wouters J, Simon JZ, Francart T (2018). Speech intelligibility predicted from neural entrainment of the speech envelope. J. Assoc. Res. Otolaryngol..

[8] Schmitt R, Meyer M, Giroud N (2022). Better speech-in-noise comprehension is associated with enhanced neural speech tracking in older adults with hearing impairment. Cortex.

[9] Calderone DJ, Lakatos P, Butler PD, Castellanos FX (2014). Entrainment of neural oscillations as a modifiable substrate of attention. Trends Cogn. Sci..

[10] Power AJ, Colling LJ, Mead N, Barnes L, Goswami U (2016). Neural encoding of the speech envelope by children with developmental dyslexia. Brain Lang..

[11] O'Sullivan JA (2015). Attentional selection in a cocktail party environment can be decoded from single-trial EEG. Cereb Cortex.

[12] Pasley BN (2012). Reconstructing speech from human auditory cortex. PLoS Biol..

[13] Marinato G, Baldauf D (2019). Object-based attention in complex, naturalistic auditory streams. Sci. Rep..

[14] Mesgarani N, Chang EF (2012). Selective cortical representation of attended speaker in multi-talker speech perception. Nature.

[15] Woldorff MG (1993). Modulation of early sensory processing in human auditory cortex during auditory selective attention. Proc. Natl. Acad. Sci. U.S.A..

[16] Ding N, Simon JZ (2012). Emergence of neural encoding of auditory objects while listening to competing speakers. Proc. Natl. Acad. Sci..

[17] Geirnaert S (2021). Electroencephalography-based auditory attention decoding: Toward neurosteered hearing devices. IEEE Signal Process. Mag..

[18] Mirkovic B, Debener S, Jaeger M, De Vos M (2015). Decoding the attended speech stream with multi-channel EEG: implications for online, daily-life applications. J. Neural Eng..

[19] Fiedler L (2017). Single-channel in-ear-EEG detects the focus of auditory attention to concurrent tone streams and mixed speech. J. Neural Eng..

[20] Das N, Bertrand A, Francart T (2018). EEG-based auditory attention detection: boundary conditions for background noise and speaker positions. J. Neural Eng..

[21] Bleichner MG, Mirkovic B, Debener S (2016). Identifying auditory attention with ear-EEG: cEEGrid versus high-density cap-EEG comparison. J. Neural Eng..

[22] Straetmans L, Holtze B, Debener S, Jaeger M, Mirkovic B (2022). Neural tracking to go: Auditory attention decoding and saliency detection with mobile EEG. J. Neural Eng..

[23] Hölle D, Meekes J, Bleichner MG (2021). Mobile ear-EEG to study auditory attention in everyday life: Auditory attention in everyday life. Behav. Res. Methods.

[24] Dasenbrock S, Blum S, Debener S, Hohmann V, Kayser H (2021). A step towards neuro-steered hearing aids: Integrated portable setup for time-synchronized acoustic stimuli presentation and EEG recording. Curr. Direct. Biomed. Eng..

[25] Cardoso VF (2021). Effect of a brain–computer interface based on pedaling motor imagery on cortical excitability and connectivity. Sensors.

[26] Kaongoen N, Choi J, Jo S (2021). Speech-imagery-based brain-computer interface system using ear-EEG. J. Neural Eng..

[27] Parbez, R. M. S. & Mamun, K. A. in *2020 2nd International Conference on Advanced Information and Communication Technology (ICAICT).* 404–409.

[28] Baek SC, Chung JH, Lim Y (2021). Implementation of an online auditory attention detection model with electroencephalography in a dichotomous listening experiment. Sensors.

[29] Marquardt DW, Snee RD (1975). Ridge regression in practice. Am. Stat..

[30] Hunter JS (1986). The exponentially weighted moving average. J. Qual. Technol..

[31] Smit AC, Schat E, Ceulemans E (2022). The exponentially weighted moving average procedure for detecting changes in intensive longitudinal data in psychological research in real-time: A tutorial showcasing potential applications. Assessment.

[32] Montoya-Martínez J, Vanthornhout J, Bertrand A, Francart T (2021). Effect of number and placement of EEG electrodes on measurement of neural tracking of speech. PLoS ONE.

[33] Narayanan AM, Bertrand A (2020). Analysis of miniaturization effects and channel selection strategies for EEG sensor networks with application to auditory attention detection. IEEE Trans. Biomed. Eng..

[34] O'Sullivan J (2017). Neural decoding of attentional selection in multi-speaker environments without access to clean sources. J Neural Eng.

[35] Haghighi M, Moghadamfalahi M, Akcakaya M, Erdogmus D (2018). EEG-assisted modulation of sound sources in the auditory scene. Biomed. Signal Process. Control.

[36] Zink R, Proesmans S, Bertrand A, Huffel SV, Vos MD (2017). Online detection of auditory attention with mobile EEG: Closing the loop with neurofeedback. bioRxiv.

[37] Miran S (2018). Real-time tracking of selective auditory attention from M/EEG: A Bayesian filtering approach. Front. Neurosci..

[38] O’Sullivan J (2017). Neural decoding of attentional selection in multi-speaker environments without access to clean sources. J. Neural Eng..

[39] Presacco, A., Miran, S., Babadi, B. & Simon, J. Z. in *2019 41st Annual International Conference of the IEEE Engineering in Medicine and Biology Society (EMBC).* 4148–4151 (IEEE). 10.1109/EMBC.2019.8857953 PMC7067200 31946783

[40] Haro S, Rao HM, Quatieri TF, Smalt CJ (2022). EEG alpha and pupil diameter reflect endogenous auditory attention switching and listening effort. Eur. J. Neurosci..

[41] Geravanchizadeh M, Roushan H (2021). Dynamic selective auditory attention detection using RNN and reinforcement learning. Sci. Rep..

[42] Ciccarelli G (2019). Comparison of two-talker attention decoding from EEG with nonlinear neural networks and linear methods. Sci. Rep..

[43] de Cheveigné A (2018). Decoding the auditory brain with canonical component analysis. Neuroimage.

[44] Geirnaert S, Francart T, Bertrand A (2022). Time-adaptive unsupervised auditory attention decoding using EEG-based stimulus reconstruction. IEEE J. Biomed. Health Inform..

[45] Han C (2019). Speaker-independent auditory attention decoding without access to clean speech sources. Sci. Adv..

[46] Wang D, Chen J (2018). Supervised speech separation based on deep learning: An overview. IEEE/ACM Trans. Audio Speech Lang. Process..

