# Supplementary material for: Validation of cost-efficient EEG experimental setup for neural tracking in an auditory attention task
Source: Sci Rep. 2023 Dec 19;13:22682. doi: 10.1038/s41598-023-49990-6 (PMC10730561; doi:10.1038/s41598-023-49990-6)
Supplement: Supplementary file 1 — Supplementary Information 1. [file 41598_2023_49990_MOESM1_ESM.docx]

**Supplementary Information**

**Dataset used for AAD simulation.**

Three datasets were used to simulate the proposed real-time AAD method. These consisted of preprocessed speech envelopes and EEG data obtained from three dichotic listening experiments involving 26 subjects and performed under laboratory conditions ^29^.

The experiments consisted of 30 trials, each of which comprised two speech segments delivered to both ears via air-conduction earphones. The speech stimuli used in the experiments were narrated in Korean by two men. In addition to the direction of attention, the order in which the stimuli were presented was also changed randomly.

The EEG signals and the speech envelopes were obtained by the same preprocessing procedure as in the actual study except for the filtering range.


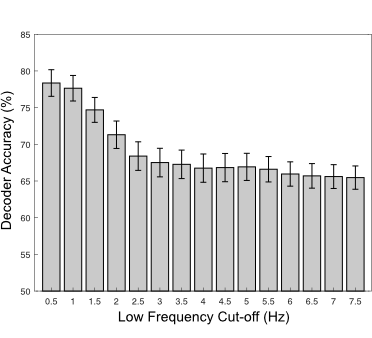


**Supplementary Figure S1.** Average decoder accuracies and standard errors of the means for different low frequency cut-off ranges across the complete dataset.

**Procedure for AAD simulation.**

Decoder models were built and evaluated with the same approach as in the *Real-time Decoder Model* session using the same three datasets. To identify the most suitable filtering range, we compared the average decoder accuracies for different low frequency cut-off values, while maintaining the high frequency cut-off at 8 Hz. The results of this AAD simulation are shown in Supplementary Fig S1. The average decoder accuracy was found to increase as the low frequency cut-off value was reduced, and the highest average decoder accuracy was achieved when the 0.5 Hz band was included (78.35 %).

**Individual Temporal Sensitivity to Changes in Spatial Attention**

A temporal sensitivity to changes in spatial attention of the listener was measured during the last four location-switching trials out of a total of 30 trials. The follow table presents the response time measured in each location-switching trial across nine subjects.

| **Trial number** | **27** | **28** | **29** | **30** |
| --- | --- | --- | --- | --- |
| Sub 1 | 15 | 5 | 1 | 5 |
| Sub 2 | 1 | - | 1 | 25 |
| Sub 3 | - | 1 | 10 | 16 |
| Sub 4 | 14 | 1 | 1 | 1 |
| Sub 5 | - | 10 | 1 | 1 |
| Sub 6 | 3 | 1 | 1 | 16 |
| Sub 7 | 1 | 2 | 17 | 18 |
| Sub 8 | 1 | 1 | 1 | 17 |
| Sub 9 | 7 | 1 | 1 | 1 |

**Supplementary Table S1.** Individual temporal sensitivity to changes in spatial attention across all subjects and four location-switching trials. Symbol ‘-‘ indicates the case in which the correct outcome was not obtained until the completion of the trial.
